# The value of coronal DWI in brainstem stroke diagnosis

**DOI:** 10.1002/ccr3.2847

**Published:** 2020-04-13

**Authors:** Prabhjot S. Bedi, Kevin J. Koch, Paul B. Lewis, Vikas K. Singh

**Affiliations:** ^1^ Department of Medicine UPMC East Monroeville PA USA; ^2^ Lake Erie College of Osteopathic Medicine OMS ‐III Erie PA USA; ^3^ Department of Radiology UPMC East Monroeville PA USA

**Keywords:** brainstem stroke, coronal sections, MRI‐DWI, posterior circulation CVA, posterior circulation stroke

## Abstract

Acute brainstem strokes can present a diagnostic challenge due to its variable clinical presentation. MRI with diffusion‐weighted (axial) imaging is highly sensitive for diagnosing ischemic lesions however even that can fail to identify early lesions in the brainstem. Combining coronal section to standard axial MRI‐DWI can facilitate early diagnosis in these cases.

## Graphical abstract

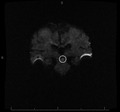

## 1 CASE

**Figure 1:**
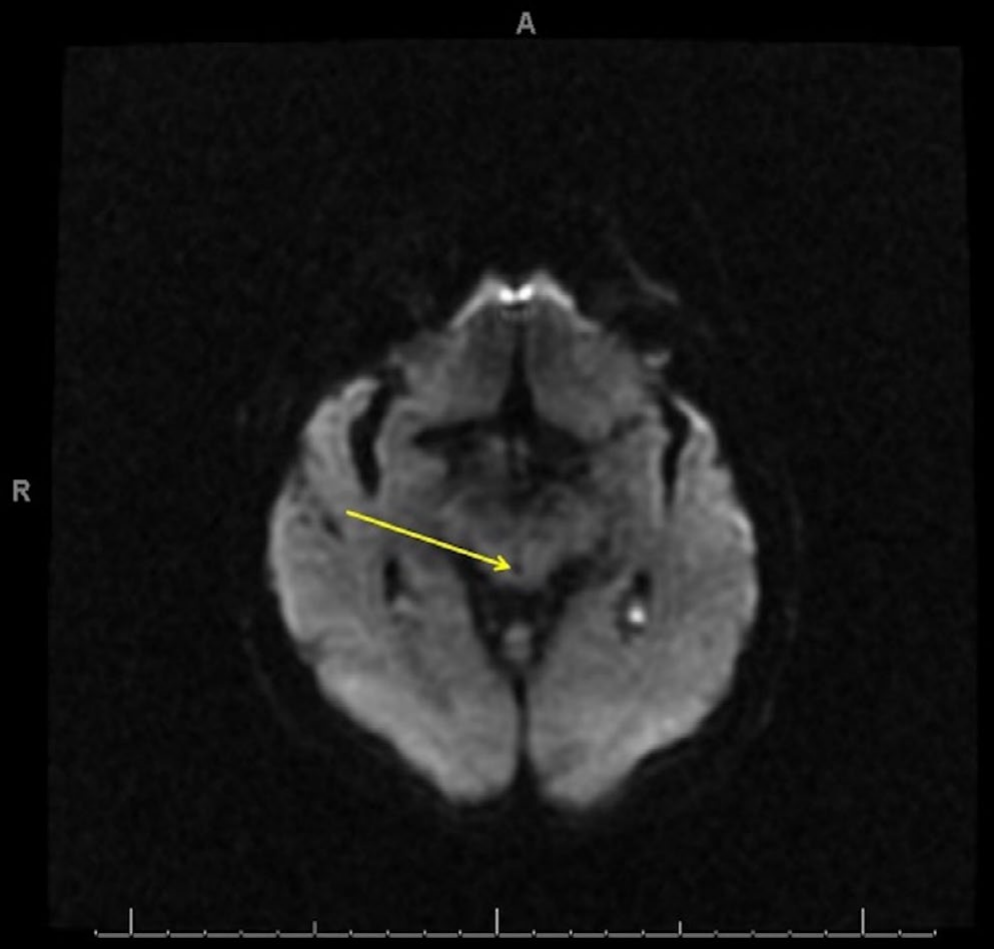
Hospital day 1 axial diffusion‐weighted image (DWI) at the level of the midbrain. No hyperintensities or other abnormal signal intensities surrounding the aqueduct of Sylvius (arrow)

**Figure 2:**
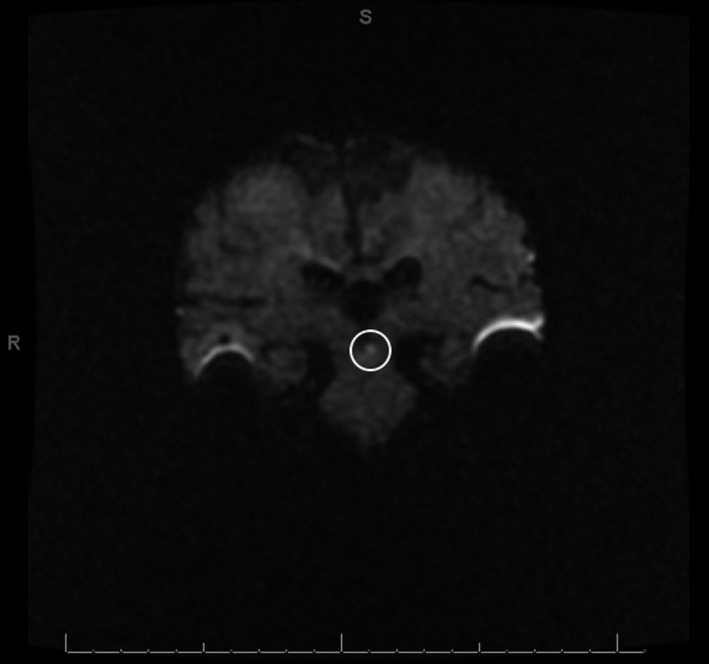
Hospital day 3 coronal DWI identifying the 3‐mm focus of restricted diffusion (circled) in the region of the left medial longitudinal fasciculus

**Figure 3:**
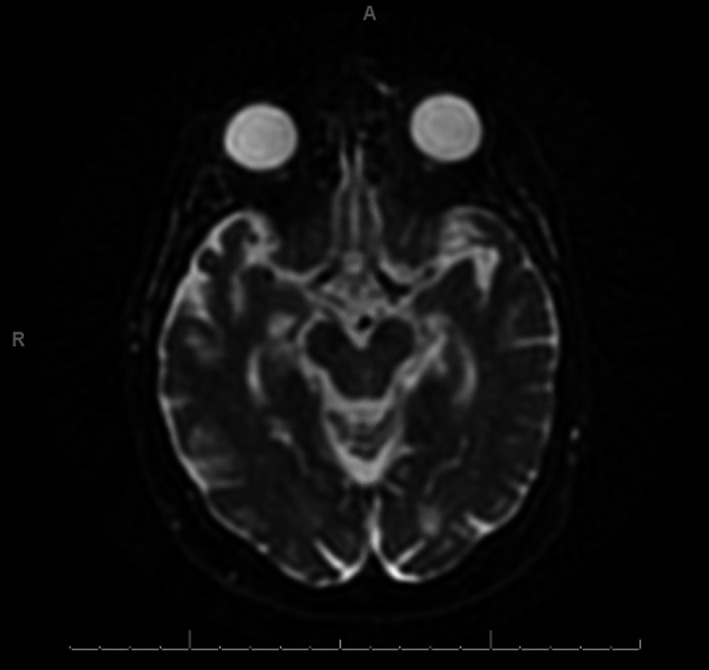
Hospital day 3 axial ADC image of the midbrain with no restricted diffusion correlating to the finding in Figure 2

**Figure 4:**
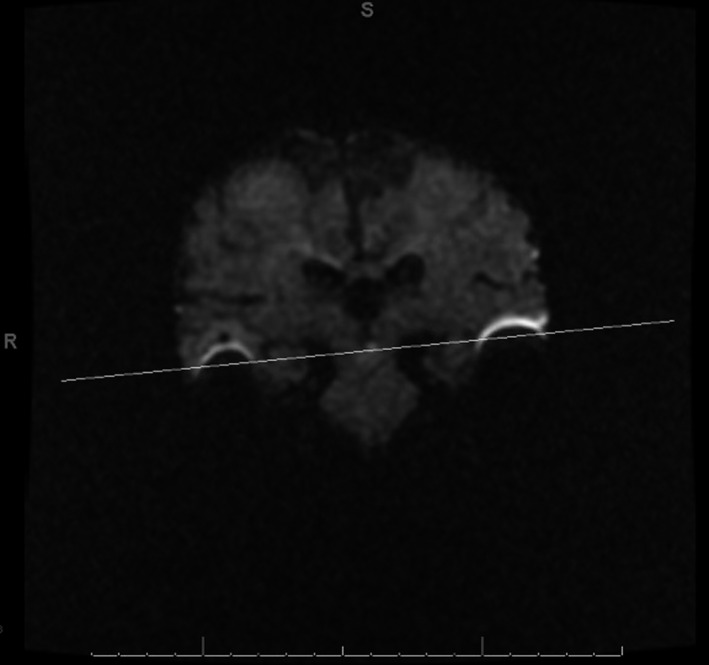
Coronal DWI with the level of the axial image in Figure 3 annotated with a white line

A 93‐year‐old man with a past medical history of benign hypertension, hyperlipidemia on a statin, atrial fibrillation on aspirin, chronic kidney disease, and hypothyroidism on levothyroxine presented on day 1 with diplopia. The clinical examination was only significant for paralysis of adduction in his left eye. CT of the head without contrast, axial MRI of the brain without contrast (Figure 1), and MRA of the brain and neck done on day 1 were unremarkable. Repeat MRI brain (a combination of standard axial and thin‐section coronal DWI) done on day 3 demonstrated a small 3 mm infarction in the periaqueductal gray matter in the midbrain, approximated around the location of the left medial longitudinal fasciculus. Ischemic lesions were only visible in coronal DWI images demonstrating its importance in detecting ischemic brain lesions (Figures 2, 3, 4).

In a case series reported by Oppenheim et al, 19% of vertebrobasilar stroke had false‐negative initial DWI study.^1^ In another single‐center study, isolated brainstem infarction was more easily identified on thin‐section coronal DWI in comparison with standard axial DWI in 35 out 155 patients (22.6%).^2^

Herein, we present a case to emphasize upon importance of using a combination of standard axial and thin‐section coronal DWI to facilitate the diagnosis of brainstem infarction. An argument can be made to include thin‐section coronal DWI of brainstem as standard protocol for all stroke workup.

## CONFLICT OF INTEREST

None declared.

## AUTHOR CONTRIBUTIONS

PSB and KJK: wrote the manuscript. PBL: procured and labeled the images. VKS: involved in literature review and edited the manuscript.

## Data Availability

Not a database study. Patient consent was obtained and available.
